# Z-DNA-induced genomic instability in the human pangenome

**DOI:** 10.21203/rs.3.rs-10129918/v1

**Published:** 2026-07-20

**Authors:** Georgios Megalovasilis, Eleftherios Bochalis, Dionysios Chartoumpekis, Karen Vasquez, Ilias Georgakopoulos-Soares

**Affiliations:** The University of Texas at Austin, Dell Pediatric Research Institute; The University of Texas at Austin, Dell Pediatric Research Institute; University of Patras; The University of Texas at Austin, Dell Pediatric Research Institute; The University of Texas at Austin, Dell Pediatric Research Institute

## Abstract

Z-DNA is a non-canonical, left-handed nucleic acid conformation with roles in gene regulation, genomic variation, and genome instability. Recent advances in long-read sequencing enable systematic interrogation of Z-DNA in repetitive and previously unresolved parts of the human genome. Leveraging the complete telomere-to-telomere reference human genome and 464 haplotype-resolved genome assemblies from individuals of multiple ethnicities, we systematically mapped Z-DNA-forming sequences and investigated their genomic and mutational landscape. We show that Z-DNA density is highly constrained across human haplotypes and superpopulations, with substantial enrichment in repetitive regions absent or unresolved in GRCh38, including centromeric, pericentromeric, and acrocentric loci. By analyzing more than 52 million variants across multiple mutation categories in the human pangenome, we show that Z-DNA loci are strongly enriched for mutational events not explained by sequence composition alone. The strongest associations were observed for small insertions, deletions, and complex structural variants. Finally, we replicated our findings using more than 250,000 *de novo* mutations. Together, these findings establish Z-DNA as a functional element of the human genome, with important implications for genome instability and human disease.

## Introduction

Z-DNA is a left-handed DNA helix that represents an alternative conformation to the canonical right-handed B-DNA (1). It is characterized by a zig-zag phosphate backbone and alternating *syn*/*anti* base conformations. Formation of Z-DNA is favored by specific sequence contexts, such as alternating purine-pyrimidine tracts of (CG)n repeats (2), whereas AT repeats are disfavored (3). Z-DNA formation is influenced by several other factors, including CpG methylation, ionic strength, torsional stress, and negative supercoiling, which either promote or hinder the transition from B-DNA to the left-handed Z conformation (4).

Z-DNA has been implicated in multiple cellular processes (5, 6). For example, Z-DNA is enriched near transcription start sites, can modulate *cis*-regulatory activity, and has functional roles in chromatin remodelling (7–12). Previous studies have also provided evidence for its role as a nucleosome-boundary element and a regulator of local chromatin accessibility (13, 14). Proteins with Z-nucleic acid-binding domains, such as ADAR1 and ZBP1, recognize and stabilize Z-DNA and Z-RNA structures (15), which has been linked to innate immune signaling, antiviral responses, and programmed cell death (8, 16, 17).

Beyond regulatory roles, Z-DNA can also promote genomic instability (15, 18–20). In both germline and somatic mutation contexts, Z-DNA-forming sequences result in elevated mutation rates across mutation categories and can contribute to the development of human diseases, including cancer (7, 20–23). However, these studies have been performed using short-read sequencing technologies, which can be error-prone and fail to adequately resolve substantial portions of the human genome that are highly repetitive (24, 25). The advent of telomere-to-telomere (T2T) genome assemblies (26) and panels of haplotype-resolved human genomes spanning diverse ancestries (27), enabled through long-read sequencing technologies, provides a complete, phased representation of these highly repetitive and complex regions that were inaccessible with older sequencing techniques. Newly resolved loci include complete centromeric and pericentromeric satellite arrays, telomeric and subtelomeric regions, the short arms of the acrocentric chromosomes, which contain ribosomal DNA arrays, and segmental duplication loci that were previously collapsed or missing from reference assemblies (26).

Several computational tools have been employed for the detection of Z-DNA-forming sequences (28–32). Guided by experimental data, we recently published ZSeeker, a fast and scalable computational tool that predicts Z-DNA-forming sequences genome-wide (23). ZSeeker has been validated against *in vivo*, *in vitro*, molecular, biochemical, biophysical studies, and structural datasets. Here, we examined the topography and genomic instability of ZSeeker-predicted Z-DNA sequences in the T2T reference human genome and across the haplotype-resolved genomes generated from the Human Pangenome Reference Consortium (HPRC).

We observed an excess of mutations overlapping predicted Z-DNA loci across multiple mutation categories, including *de novo* mutations. We further evaluated how this enrichment varied by mutation class, variant length, genomic context, and other parameters, providing insights into the relationship between Z-DNA-forming sequences and genetic instability. We report that Z-DNA-forming sequence density exhibits a 214 bp/Mb increase in the T2T assembly compared to the gapped human assembly. Across more than 52 million pangenome variants, mutations are significantly enriched near Z-DNA, with a 37.9-fold increase in complex 6–50 bp deletions. Such enrichment is not identified genome-wide, but is constrained to genic euchromatic regions. Analysis of more than 250,000 *de novo* mutations further validates this enrichment, indicating that elevated mutability at Z-DNA-forming sequences is an intrinsic property and not a consequence of selection.

With these results, we conclude that Z-DNA-forming sequences are pervasive and evolutionarily constrained features of the human genome that are strongly associated with elevated genetic instability, with potential implications for genome evolution, inherited variation, and disease-associated mutagenesis.

## Results

### Characterization of Z-DNA predictions across human haplotypes

Using the T2T (26) reference human genome from the CHM13hTERT cell line (T2T-CHM13), as well as the 464 T2T human haplotypes provided by the second release of the HPRC (27), we examined the distribution and population-wide conservation of Z-DNA-forming sequences across human chromosomes. The set of haplotypes comprised allelic profiles from diverse ancestries, across five super-populations: African (AFR, *n* = 140); East Asian (EAS, *n* = 102); American (AMR, *n* = 88); South Asian (SAS, *n* = 72); and European (EUR, *n* = 62).

**Figure 1:**
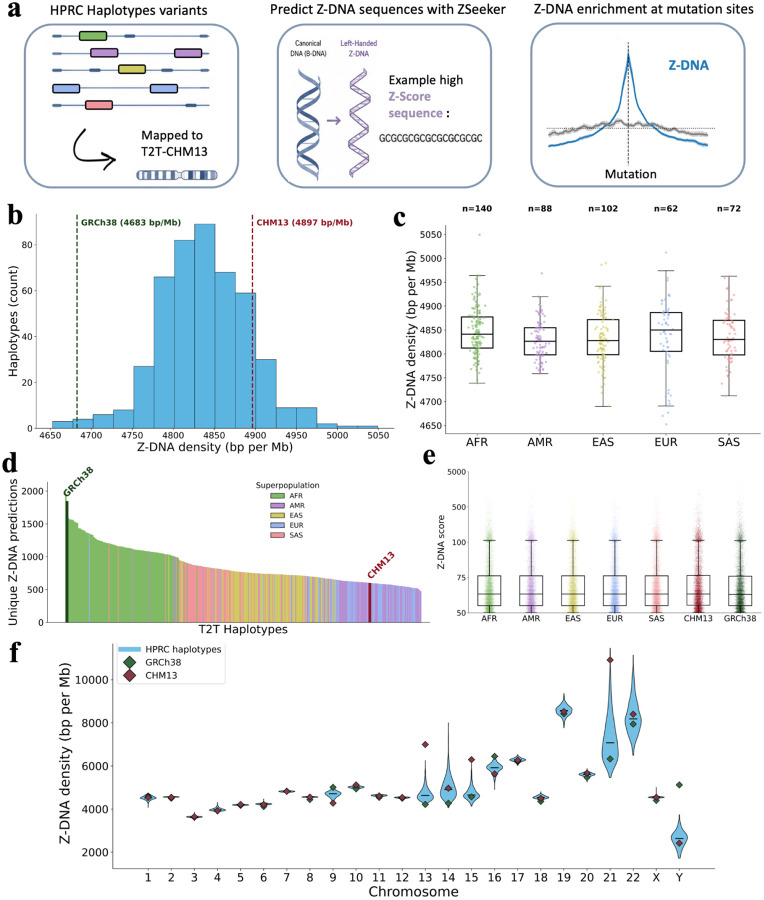
Genome-wide Z-DNA density is tightly constrained across human haplotypes. **a.** Simplified schematic illustration of our approach to evaluate Z-DNA density and impact on genetic instability. **b.** Distribution of Z-DNA density (bp/Mb) across 464 HPRC haplotypes and the two human reference genomes; T2T-CHM13 highlighted in dark red and GRCh38 in green. **c.** Violin plots depicting Z-DNA density stratified by superpopulations. **d.** Bar plots showing haplotype-specific Z-DNA sequence count for all 466 genomes, colored by superpopulation and reference genomes. **e.** Box plots of Z-DNA score distributions for the same categories. Each category was randomly sampled to include 200,000 Z-DNA-forming sequences. **f.** Chromosome-level Z-DNA density across haplotypes visualized with violin plots.

Using the approach outlined in Fig. 1a, we found a Z-DNA density of 4,897 bp/Mb for T2T-CHM13, compared to 4,683 bp/Mb for GRCh38 and a mean Z-DNA density of 4,837 bp/Mb across the 464 HPRC haplotypes (Fig. 1b). The reduced Z-DNA density in GRCh38 likely reflects the unresolved repetitive regions absent from this assembly and errors that were fixed due to long-read sequencing. Z-DNA density was stable across superpopulations (Fig. 1c), whereas the number of haplotype-specific Z-DNA predicted sequences varied substantially. AFR assemblies exhibited the highest counts of haplotype-specific sequences (up to 2,035), while AMR and EUR haplotypes generally showed lower counts (Fig. 1d). CHM13 contained comparatively few haplotype-specific Z-DNA sequences (n = 601). At the same time, GRCh38 featured a higher number (n = 1,848), consistent with its multi-donor assembly composition.

ZSeeker provided a Z-DNA score metric for each prediction, quantifying the propensity of a sequence to adopt the Z-conformation (23). Comparing the two reference genomes and the five superpopulations (Fig. 1e), we observed highly similar score distributions across all groups, with nearly identical distributions. Predicted Z-DNA sequence lengths followed the same pattern (**Suppl. Figure 1a**). When stratifying Z-DNA density by chromosome (Fig. 1f), we observed chromosome-specific differences in median density that were strongly associated with chromosomal GC content (Spearman *ρ* = 0.74, *p* = 7.035 × 10^−5^) (**Suppl. Figure 1b**). Notably, acrocentric chromosomes were among those with the most prominent differences in Z-DNA density between the two reference genomes. Acrocentric chromosomes 13 and 21 exhibited the highest difference, with increases in densities upwards of 2,780 and 4,589 bp/Mb, respectively (Fig. 1f). These patterns likely reflect the T2T resolution of repetitive rDNA- and satellite-rich regions on the acrocentric short arms that are absent or incomplete in GRCh38.

We also asked whether, in trio-phased assemblies, the autosomes exhibit a consistent paternal-maternal shift in Z-DNA signal that would indicate a parent-of-origin effect (**Suppl. Figure 1c**). Across 128 individuals, the median paternal-maternal difference was + 8.6 bp/Mb, with no significant paired difference observed between haplotypes (Wilcoxon signed-rank *p* = 0.082), indicating no systematic parent-of-origin bias in autosomal Z-DNA density. These results suggest a rather constrained Z-DNA density profile for human haplotypes, without significant ancestry or parental differences.

### Z-DNA is enriched at transcriptionally active genomic loci in the CHM13 human genome

**Figure 2:**
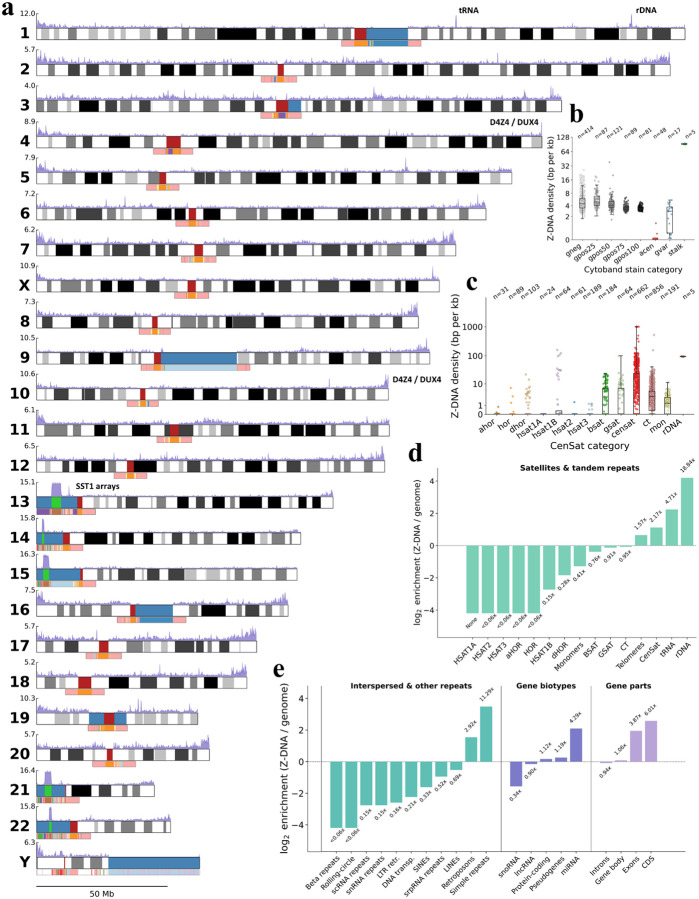
Z-DNA-forming sequences are non-uniformly distributed across the human genome. **a.** Karyoplot of Z-DNA density (purple upper track, 150 kb bins, y-axis featuring Z-DNA density in kb / 150 kb) across all chromosomes of the T2T-CHM13 assembly. Cytobands (middle tracks) are shown with standard Giemsa stain coloring, and CenSat annotation categories are featured in the lower track. Manual text annotations indicate strong local Z-DNA signal. **b.** Z-DNA density (bp per kb, log_2_-scaled y-axis) across cytoband stain categories. Boxplots show median and interquartile range; individual cytoband intervals are overlaid as jitter points. **c.** Z-DNA density (bp per kb, log_10_-scaled y-axis) across CenSat annotation categories, displayed as in b. **d-e.** Log_2_ Z-DNA enrichment relative to genome-wide Z-DNA density across 34 annotation categories: satellites and tandem repeats (d.); interspersed and other repeats; gene biotypes; and gene substructures (e). Fold-change values are labeled above each bar. Repeat abbreviations are presented in **Supplementary Table 1**.

Genome-wide ideogram visualization (Fig. 2a) of the CHM13 genome revealed that Z-DNA is not uniformly distributed and is most enriched near the rDNA regions of acrocentric chromosomes, consistent with our previous findings (23). We also observed discrete Z-DNA enrichment peaks at additional loci, including tRNA clusters, SST1 satellite regions, and D4Z4 repeat arrays (Fig. 2a). Quantification of Z-DNA density across cytoband stain categories showed that euchromatic cytobands (gneg and gpos25) exhibited higher density than heterochromatic bands (gpos75 and gpos100) (median 4.68 vs. 3.45 bp/kb; Mann-Whitney U, *q* = 2.9 × 10^−24^; Cliff’s *δ* = 0.52; Fig. 2b). Centromeric regions (acen) were almost entirely depleted of Z-DNA relative to non-centromeric cytobands (median 0.0 vs. 4.1 bp/kb; *q* = 7.2 × 10^−31^; Cliff’s *δ* = −0.999). The highest densities were observed in stalk regions, which correspond to nucleolar organizer/rDNA loci (median 90.8 bp/kb; *q* = 1.1 × 10^−4^ vs. all other cytobands). Subsequent analysis of centromeric satellite regions confirmed and extended these results (Fig. 2c). Examining sex chromosome-specific regions (**Suppl. Figure 1d**) Z-DNA density varied, with pseudoautosomal regions (PAR) showing the highest median density (12,828 bp/Mb; n = 4), followed by ampliconic (median 5,077 bp/Mb; n = 7) and X-degenerate regions (median 4,112 bp/Mb; n = 8). Satellite, centromeric, and heterochromatic regions exhibited lower densities, consistent with the depletion pattern observed earlier.

Next, we quantified Z-DNA enrichment across 34 genomic annotation categories relative to the genome-wide Z-DNA density (Fig. 2d–e). rDNA arrays showed the strongest enrichment of any category (18.84-fold), followed by tRNA arrays (4.71-fold) and CenSat repeats (**Supp. Table 1)** (2.17-fold). Within CenSat repeats, HSAT1A (no detectable Z-DNA), HSAT2, HSAT3, and HOR and AHOR were depleted (< 0.01–0.15-fold), whereas BSAT, GSAT, and CT regions showed moderate to minimal depletion (0.76–0.95-fold). Among interspersed repeat classes, all major transposable element families (LTR, DNA transposons, SINEs, and LINEs) were depleted (0.16–0.69-fold), whereas simple repeats (11.29-fold) and retroposons (2.92-fold) were strongly enriched. For gene biotypes, miRNA genes showed the highest enrichment (4.29-fold), while protein-coding genes (1.12-fold) and pseudogenes (1.19-fold) were also enriched. Among gene substructures, coding sequences (6.01-fold) and exons (3.87-fold) were strongly enriched, gene bodies were near baseline (1.06-fold), and introns were slightly depleted (0.94-fold).

We conclude that Z-DNA is enriched in euchromatic, transcription-associated loci, largely excluded from major heterochromatic repeat compartments, and highly enriched at transcriptionally active loci and rDNA genes.

### Z-DNA is associated with higher mutagenicity across most mutation categories

To directly assess whether Z-DNA loci represent localized hotspots of genomic instability, we next examined mutation burden across haplotypes obtained from the deconstruction of the HPRC pangenome graph. We classified 52 million mutations into 21 mutation categories (based on their type, size, and breakpoints in the case of large variants) from single-nucleotide variants (73.5% of events) to complex indels (3.2%; **Suppl. Figure 1e**). To determine whether Z-DNA-forming sequences are preferential sites of mutation, we examined mutational enrichment across variant categories using two complementary approaches.

**Figure 3:**
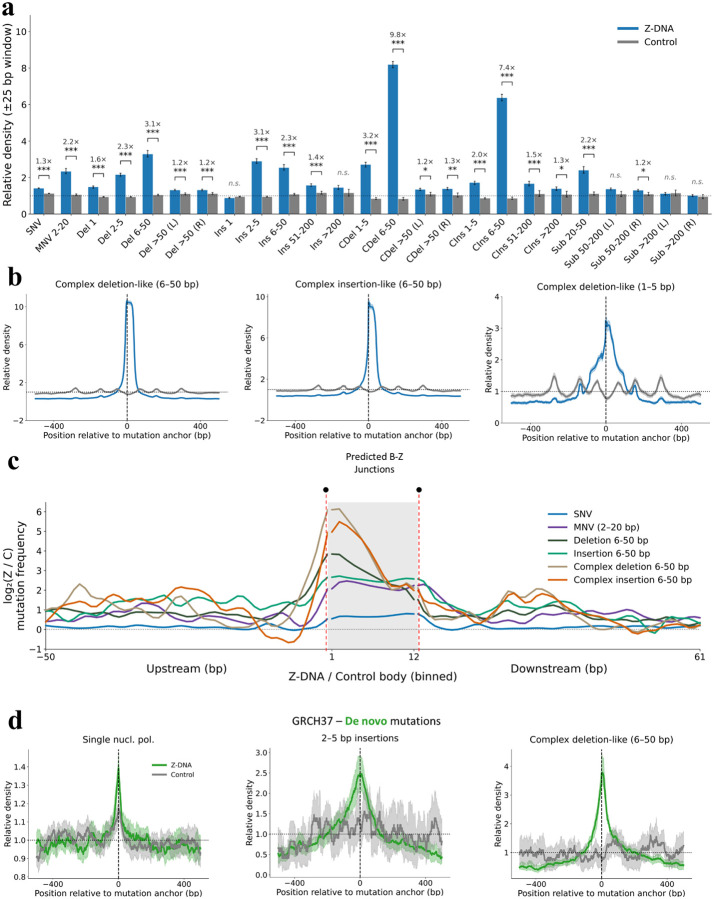
Predicted Z-DNA-forming sequences feature distinct profiles around mutational hotspots. **a.** Bar chart of relative densities of Z-DNA (blue) predictions versus Controls (gray) around a 50 bp window of 21 different mutation categories from the HPRC-CHM13 variant dataset. For dual-anchor strata, both breakpoints are shown (L for left and R for right). Stars denote Wilcoxon signed rank test significance after Benjamini-Hochberg FDR correction, while error bars indicate 95% chromosome block-bootstrap confidence intervals. **b.** Z-DNA metaprofiles for the three most highly Z-DNA-enriched mutation categories from the HPRC-CHM13 variant dataset. For each type, Z-DNA overlap (blue) and matched control overlap (gray) were calculated in a ±500 bp window around the mutation point and normalized over the window mean to give relative density. Shaded bands show 95% chromosome-block bootstrap confidence intervals (CIs). The dashed vertical line marks the mutation position (offset = 0) and the horizontal one indicates the normalized baseline. **c.** Z-DNA-centered metaprofiles for different mutation categories. Mutation frequencies were calculated across 50 bp upstream and downstream regions and across the Z-DNA/control interval body (rescaled into equal bins due to varying Z-DNA lengths). The gray central region represents the Z-DNA/control body.

To test whether Z-DNA-forming sequences are associated with elevated mutagenesis, we computed the relative density of these variants within the vicinity of predicted Z-DNA intervals versus composition-matched control regions (Fig. 3a). Z-DNA loci displayed a pronounced enrichment peak centered on the mutation site, compared to composition-matched control regions, which remained largely flat and close to the normalized baseline (Fig. 3b). The majority of mutation categories showed significant enrichment near Z-DNA (Wilcoxon signed-rank test, *q* < 0.01 for 18 out of the 21 mutation categories). The strongest effects were observed for small- and medium-sized complex indels (Fig. 3b): complex deletions of 6–50 bp (9.8-fold), complex insertions of 6–50 bp (7.4-fold), and complex deletions of 1–5 bp (3.2-fold).

Beyond complex events, most simple deletions and insertions displayed Z-DNA enrichment (1.2- to 3.1-fold), as did multi-nucleotide variants and small substitutions (2.2-fold). SNVs exhibited a more modest enrichment (1.3-fold), but when all six substitution categories were examined, T > C transitions showed the strongest effect (1.45-fold), followed by T > G, T > A, and C > T (1.23–1.25-fold) (**Suppl. Figure 2a**). Conversions C > A and C > G displayed the weakest enrichment values (1.07–1.09-fold; **Suppl. Figure 2a**). Finally, a few categories showed no significant enrichment (**Suppl. Figure 2b**; *q* > 0.01), two representing larger structural variants: insertions and substitutions greater than 200 bp (0.98–1.20-fold) and 1 bp insertions (0.93-fold).

To confirm that the observed enrichment reflects the Z-DNA interval itself rather than broader sequence context, we divided each Z-DNA sequence into length-normalized bins and compared the mutation frequencies across the bins against those of the composition-matched and length-matched control sequences using Z-DNA-centred metaprofiles (Fig. 3c). For the six representative mutation categories, log_2_(Z-DNA/Control) mutation frequency was highly elevated within the Z-DNA sequence, and specifically peaked near the predicted B-Z junctions and decayed toward baseline in the flanking regions. Complex deletions of 6–50 bp exhibited the strongest enrichment (37.9-fold), followed by complex insertions of 6–50 bp (28.0-fold), simple deletions of 6–50 bp (9.7-fold), simple insertions of 6–50 bp (6.0-fold), and MNVs (4.7-fold), while SNVs displayed modest enrichment (1.6-fold) and 1 bp insertions (**Suppl. Figure 2c**) featured a mild depletion (0.7-fold; 95% CI: 0.6–0.8). All comparisons were statistically significant on chromosome-level frequencies (one-sided Wilcoxon signed-rank test, *q* = 6 × 10^−8^). These results indicate that most mutation classes are preferentially clustered near predicted B-Z junctions.

### Z-DNA mutagenicity correlates with variant size, Z-DNA score, and allele frequency

**Figure 4:**
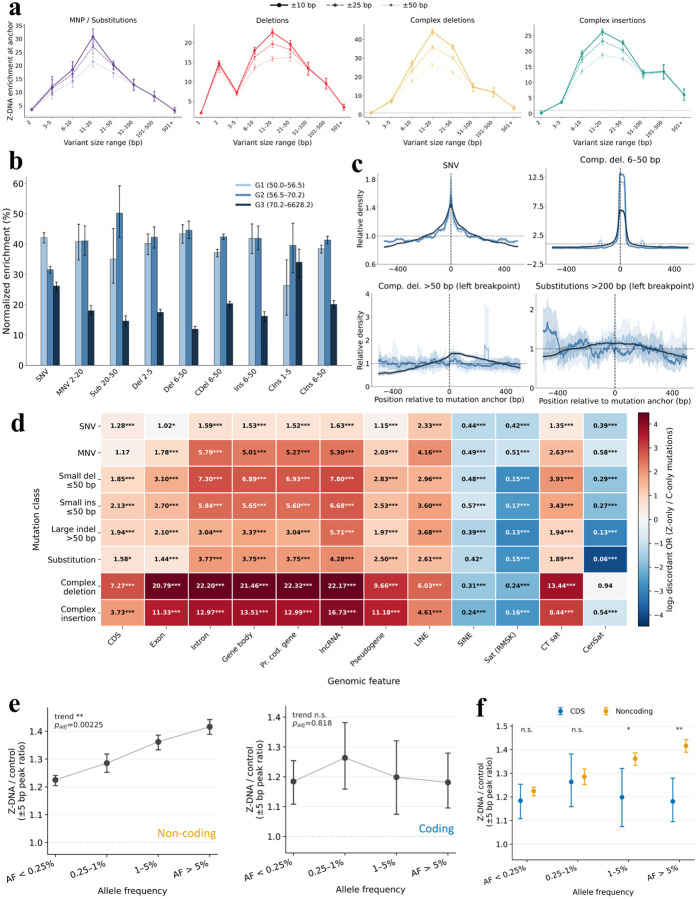
Z-DNA-associated mutagenesis depends on variant size, Z-tract score, localization and allele frequency. **a.** Z-DNA enrichment as a function of variant size. Enrichment is the ratio of the local Z-DNA base fraction (within ±10, ±25, or ±50 bp of the mutation anchor) to the genome-wide Z-DNA fraction (0.48%). Points: chromosome-block bootstrap means; error bars: 95% CIs. **b.** Score-stratified Z-DNA enrichment for representative mutation strata. Predicted Z-DNA regions were divided into three equal-count groups by Z-DNA score: G1 (50.0–56.5, light blue), G2 (56.5–70.2, medium blue), and G3 (70.2–6628.2, dark blue). Bars show each score group’s contribution to the total excess Z-DNA-associated signal within each mutation stratum, normalized to 100% across G1-G3. Error bars indicate 95% chromosome-block bootstrap confidence intervals. Pairwise differences between score groups were assessed using chromosome-level Wilcoxon signed-rank tests with BH-FDR correction. **c.** Score-stratified mutation-anchored metaprofiles for SNVs, complex deletions 6–50 bp, complex deletions >50 bp, and substitutions >200 bp. For dual-anchor classes, the left breakpoint is shown. Lines show self-normalized relative Z-DNA density across ±500 bp from the mutation anchor for each Z-DNA score group; shaded bands indicate 95% chromosome-block bootstrap confidence intervals. The dashed vertical line marks the mutation anchor and the dotted horizontal line marks the normalization baseline. **d.** Discordant odds ratio heatmap (variants uniquely proximal to Z-DNA vs. uniquely proximal to matched control) for each mutation class across genomic features. Anchors within ±10 bp of a Z-DNA interval and of a length-/composition-matched control interval were scored per variant; the McNemar OR contrasts the two. Color: log_2_ OR (red, enriched near Z-DNA; blue, depleted). Stars indicate BH-FDR-adjusted significance (*: *q*< 0.05, **: *q* < 0.01, ***: q < 0.001). Features overlap, so ORs are not additive across columns. Features failing the Z/control matching were excluded. **e.** Z-DNA/control density ratio at SNV sites (±5 bp metaprofile peak) as a function of within-HPRC allele frequency, in noncoding (left) and coding (right) sequences. Points: chromosome-block bootstrap means; error bars: 95% CIs. Trend q: BH-FDR-adjusted bootstrap test of the slope across AF bins. **f.** Direct comparison of the ±5 bp Z-DNA/control peak ratio between CDS (blue) and noncoding (orange) per allele-frequency bin. Error bars: 95% bootstrap CIs. Stars: BH-adjusted between-region test (*n.s*.: not significant, *: *q* < 0.05, **: *q* < 0.01).

To test whether Z-DNA-associated mutagenesis depends on variant size, we determined Z-DNA enrichment within a window around mutation anchors. Windows of ± 10, ±25, and ± 50 bp were evaluated to assess the spatial concentration of the effect. Z-DNA enrichment levels were strongly linked to the variant size. The four indel and complex variant categories showed the strongest Z-DNA enrichment at intermediate event sizes. Complex deletion-like variants were the most enriched, peaking at 44.1-fold within ± 10 bp for the 11–20 bp length, followed by complex insertion-like variants (26.2-fold) and deletions (22.7-fold), both of which also peaked at the 11–20 bp length (Fig. 4a). Insertions showed the highest enrichment at 2 bp length (17.3-fold) but also featured a second, broader maximum at 51–100 bp length (16.8-fold; **Suppl. Figure 2d**). MNVs showed the highest enrichment (30.9-fold) at 11–20 bp. Across all categories, enrichment attenuated as the window widened, with the ± 25 and ± 50 bp curves falling consistently below ± 10 bp, indicating that the association is concentrated at the variant site rather than reflecting a broader regional effect.

We next asked whether Z-DNA-associated mutagenesis varies with predicted Z-DNA score. For this, we partitioned the predicted Z-DNA loci in T2T-CHM13 into three equal-count terciles of increasing Z-DNA score (G1 < G2 < G3), which also differed in Z-DNA tract length, with median lengths increasing from 30 bp in G1 to 44 bp in G2 and 61 bp in G3. We then recomputed mutation-anchored enrichment profiles separately for each score group and quantified each score group’s contribution to the total excess Z-DNA-associated signal. Across many mutation classes, particularly MNVs, small and intermediate indels and complex indels, and short substitutions, the lower- and intermediate-score groups carried most of the excess enrichment signal (Fig. 4b), whereas G3 showed significantly reduced signal relative to both G1 and G2 (chromosome-level pairwise Wilcoxon signed-rank tests, *q* < 0.05). The strongest examples were complex deletions of 6–50 bp, complex insertions of 6–50 bp, 6–50 bp deletions, 2–5 bp deletions, 6–50 bp insertions, MNVs, and 20–50 bp substitutions, for which G1 and/or G2 showed substantially higher enrichment than G3. SNVs showed a distinct monotonic pattern (Fig. 4c), with excess signal decreasing from G1 to G2 to G3 (mean relative density 1.70, 1.52, and 1.43, respectively; all pairwise *q* < 0.001). In contrast, larger-event classes showed weaker and less consistent score-dependent behavior, with some categories showing no significant pairwise differences and others showing higher enrichment in G3. Representative metaprofiles (Fig. 4c) illustrate these regimes: a sharp score-ordered central peak for SNVs, strong G1/G2-over-G3 separation for complex 6–50 bp deletions, and broader, less consistently ordered profiles for larger events such as complex deletions > 50 bp and substitutions > 200 bp. One possible explanation is that higher-scoring Z-DNA regions also tend to be longer, and very long predicted Z-DNA tracts may be less likely to form or maintain stable Z-DNA structures in vivo.

### Z-DNA is enriched around mutations in genic and euchromatic compartments

To examine differences in Z-DNA-associated genomic instability across genomic sub-compartments, we compared, for each mutation category and genomic feature, the odds that a variant lies within ± 10 bp of a Z-DNA interval versus a length- and composition-matched control interval using a discordant McNemar odds ratio (Fig. 4d). Features were tested independently, so a mutation could contribute to multiple genomic-feature cells.

Out of the 96 feature-mutation comparisons (Fig. 4d), 94 were statistically significant (*q* < 0.05): 71 showed enrichment of mutations near ZDNA relative to matched controls (OR > 1), whereas 23 showed depletion (OR < 1). Enrichment was concentrated in genic compartments and LINEs, while depletion was concentrated in SINEs, RepeatMasker satellites, and centromeric satellites regions. Complex deletions showed the strongest associations in exons, introns, gene bodies, protein-coding genes, and lncRNAs (OR = 20.8–22.3), with lower but still marked enrichment in coding sequences and pseudogenes. Complex insertions were also strongly enriched across these genic features, particularly in lncRNAs, gene bodies, introns, protein-coding genes, and exons (OR = 11.3–16.7; *q* < 0.05). SNVs showed comparatively modest enrichment in genic regions (OR = 1.02–1.63), although stronger SNV enrichment was observed in LINEs (OR = 2.33). In contrast, SINEs and RepeatMasker satellites showed consistent depletion across all mutation classes (OR = 0.13–0.57), and CenSat regions were also depleted for most mutation classes (OR = 0.06–0.58), with complex deletions in CenSat being the only non-significant depletion-like comparison (OR = 0.94). CTs were a clear exception among CenSat annotations, showing significant Z-DNA-associated enrichment across all mutation classes (OR = 1.35–13.44).

We also assessed whether their Z-DNA association varied within-HPRC allele frequency, separately for coding and noncoding sequences. In noncoding sequences, the ± 5 bp Z-DNA/control peak ratio at SNV sites rose monotonically with allele frequency, from 1.23-fold for very rare alleles to 1.42-fold for those with a frequency above 5% (*q* = 0.002; Fig. 4e–f), and this positive trend was stable across allele-number thresholds (counts of haplotypes harboring info for a specific allele) from 10 to 400. Coding SNVs showed no significant trend. Higher-frequency SNVs are therefore more concentrated near Z-DNA in noncoding sequences specifically, highlighting a compartment-specific relationship.

Together, these results indicate that SNVs with higher within-HPRC allele frequencies are preferentially concentrated near Z-DNA-forming sequences in noncoding regions, whereas coding SNVs show no comparable frequency-dependent pattern. This suggests a compartment-specific relationship between Z-DNA and standing genetic variation, potentially reflecting differences in mutational processes, tolerance, and/or constraint between coding and noncoding sequences.

#### Z-DNA mutational enrichment is detectable in de novo mutations

**Figure 5:**
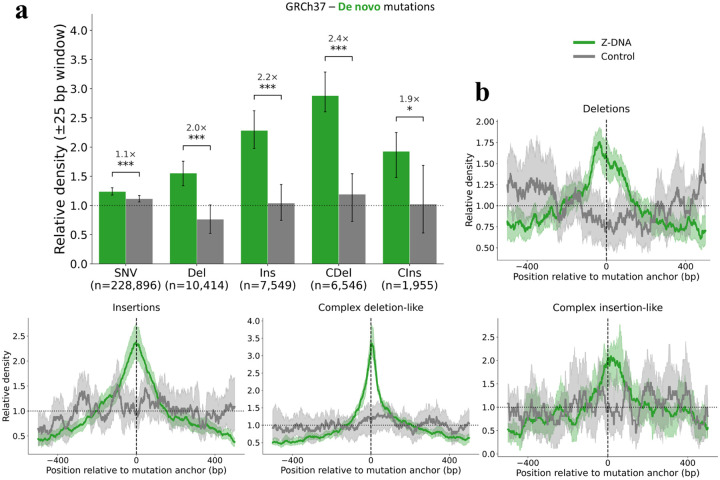
*De novo* mutations are enriched at predicted Z-DNA loci. **a.** Relative density of *de novo* mutations within ±25 bp of predicted Z-DNA regions (green) versus composition-matched control regions (grey), grouped by mutation class. Bars show the window-averaged relative density (self-normalized to the ±500 bp profile mean); error bars indicate 95% chromosome block-bootstrap confidence intervals. Fold enrichment and significance are annotated from one-sided chromosome-block bootstrap tests after BH-FDR correction (*: *q* < 0.05, **: *q* < 0.01, ***: *q*< 0.001). Sample sizes are shown below each class. MNVs (n = 481) were excluded due to insufficient counts (<500). **b.** Per-class relative-density metaprofiles across the ±500 bp window centered on the mutation anchor. Shaded bands represent 95% chromosome block-bootstrap confidence intervals. The dashed vertical line marks the mutation site; the dotted horizontal line marks the self-normalized baseline.

Population variants reflect genetic variation that has persisted in human populations and has therefore been exposed to the effects of natural selection. To test whether Z-DNA-associated mutagenesis is detectable before selective forces can eliminate or retain variants, we performed the same analysis on 255,841 *de novo* WGS-derived mutations from denovo-db (33) (Fig. 5a) on the GRCh37 reference genome. *De novo* SNVs (*n* = 228,896; **Suppl. Figure 2e**) showed significant Z-DNA enrichment (1.1-fold; one-sided, *q* = 1.5 × 10^−4^), comparable to the population-level HPRC signal (1.3-fold). Simple deletions (*n* = 10,414; 2.0-fold) and insertions (*n* = 7,549; 2.2-fold) were enriched, and the strongest effects were again observed for complex events: complex deletion-like (*n* = 6,546; 2.4-fold) and complex insertion-like (*n* = 1,955; 1.9-fold) variants (all *q* values < 0.02). Across all categories, the matched control profiles remained close to the normalized baseline (Fig. 5b).

## Discussion

In this study, we systematically mapped Z-DNA-forming sequences across the T2T reference human genome and in 464 haplotype-resolved human genome assemblies and demonstrated that Z-DNA imposes a consistent and substantial mutational burden in the human genome. Using two complementary analytical approaches, we show that mutations cluster at Z-DNA loci across all major variant classes, with the strongest enrichment observed for small deletions between 6 and 50bp, complex insertions and deletions, reaching a ~ 10-fold enrichment for complex deletions. Importantly, this enrichment was recapitulated in *de novo* mutations, indicating that it reflects an intrinsic mutational propensity of Z-DNA-forming sequences rather than selective retention or elimination of variants over evolutionary time.

Although prior computational studies have established a clear link between Z-DNA and genomic instability, these analyses were constrained by short-read sequencing technologies, which exhibit elevated error rates at non-B DNA motifs and fail to resolve the highly repetitive regions of the human genome (7, 21, 22, 34–36). By using high-quality long-read sequencing datasets and complete T2T genome assemblies, we confirm prior findings on Z-DNA-induced genomic instability, while resolving its mutational footprint across repetitive regions that were systematically inaccessible to previous analyses. The elevated Z-DNA density we report in T2T-CHM13 relative to GRCh38, attributable to newly resolved centromeric, pericentromeric, and rDNA regions, underscores the importance of complete genome assemblies for accurately characterizing non-B DNA biology. Because *de novo* mutations have not been subject to selective pressures, the enrichment at Z-DNA loci demonstrates that the elevated mutational rate at these sequences, at least in part, is an intrinsic structural property rather than a signature imposed by evolutionary filtering of pre-existing variation.

The mutagenic processing of Z-DNA is mediated by the mismatch repair complex MSH2-MSH3 and the nucleotide excision repair endonuclease ERCC1-XPF, which is recruited to Z-DNA in an MSH2-MSH3-dependent, NER-independent manner and generates strand breaks near Z-DNA-forming regions (19). Our results are consistent with this model; mutational enrichment is tightly concentrated at the mutation site and attenuates rapidly at wider windows, implying that the mutagenic effect originates at the structure itself. The extreme enrichment of complex deletion-like events further supports a double-strand break intermediate, in line with prior mammalian cell studies in which Z-DNA-forming sequences generated large-scale deletions in the vast majority of the mutants identified (18).

The prevalence of genome-wide Z-DNA density in the human population, despite its genomic mutagenicity, suggests that Z-DNA-forming sequences may be subject to competing evolutionary pressures. On one hand, Z-DNA may promote genetic instability, and on the other, its enrichment near transcriptionally active and regulatory regions implies potential functional constraint (37). This is consistent with a model in which Z-DNA contributes both to genome regulation and to sequence-intrinsic mutational vulnerability.

Our findings carry direct implications for human disease etiology. For instance, gross chromosomal rearrangements, a hallmark of cancer genomes, have previously been linked to Z-DNA at breakpoints of oncogenes including *BCL-2* and *c-MYC*. Notably, we further detected Z-DNA enrichment at the SST1 and the D4Z4 arrays. SST1 has been established as a common breakpoint of human Robertsonian translocations on the acrocentric short arms (38). Contraction of the D4Z4 repeat has been associated with facioscapulohumeral muscular dystrophy (39). The colocalization of predicted Z-DNA-forming sequences with these pathologically relevant arrays nominates Z-DNA as a candidate contributor to their structural fragility. This hypothesis, however, requires further future work.

The extreme enrichment of complex structural variants at Z-DNA loci reported here, reaching 34.3-fold at Z-DNA loci, extends this association to a genome-wide scale and into previously unresolved repetitive regions, underscoring the need for future studies examining Z-DNA-associated instability in the context of disease-relevant mutations. Moreover, the extension of this enrichment to *de novo* mutations confirms that it reflects an intrinsic mutational propensity rather than the selective retention of cancer-associated genetic instability.

Future work is required to dissect the mechanisms through which Z-DNA promotes genomic instability and the implications for human disease aetiology. Ultimately, characterizing the contribution of Z-DNA-associated mutagenesis to disease development, particularly in the repetitive and regulatory regions now accessible through T2T assemblies, may open new avenues for understanding the sequence-intrinsic origins of human genetic disease.

## Methods

### Prediction of Z-DNA sequences with ZSeeker

Z-DNA sequences were predicted using the ZSeeker software (v1.8) (23) across all 464 human HPRC haplotypes, and the T2T-CHM13, the GRCh38, and the GRCh37 reference genomes. Scoring and penalty settings were left at their default values. ZSeeker outputs non-overlapping Z-DNA predictions per chromosome, and we have used these predictions without reducing overlapping regions to one Z-DNA site.

### Z-DNA density and enrichment

Z-DNA density was defined as the number of ZSeeker-predicted Z-DNA bases per length of the examined region. For Z-DNA enrichment, two different estimations were employed based on the desired question/analysis. First, density enrichment: the Z-DNA base density within a given interval, annotation class, or local window divided by the genome-wide Z-DNA base density. Second, control-referenced enrichment: for each mutation anchor, Z-DNA coverage at positions across a ± 500 bp window was computed and normalized to the window mean, yielding a unitless relative density; the same was applied to the composition- and length-matched control intervals. Enrichment was quantified as the ratio of the Z-DNA to control relative density, or as the discordant odds ratio of mutations uniquely proximal to Z-DNA versus uniquely proximal to control. Window sizes, statistical tests, and multiple-testing corrections are specified per analysis in the corresponding Results sections and/or figure legends.

### Mutational analysis

Variant sites were extracted from the HPRC v2.0 Minigraph-Cactus VCF projected onto T2T-CHM13. We used a custom Python parser that processes the VCF record by record and classifies each reference/alternate allele pair from the two allele lengths in a fixed precedence. Writing L_ref and L_alt for the reference and alternate lengths: a pair with L_ref = L_alt = 1 was a SNV, equal lengths greater than 1 were a MNV when L ≤ 20 bp and a substitution when L > 20 bp, L_ref < L_alt was an insertion when L_ref = 1 and otherwise a complex (insertion-like) event and L_ref > L_alt was a deletion when L_alt = 1 and otherwise a complex (deletion-like) event. Each variant was reduced to a single 1-bp anchor used for all downstream proximity and metaprofile analyses: the variant base for SNVs, the reference-allele midpoint for MNVs and substitutions, the left breakpoint (first changed base) for deletions and deletion-like complex events, and the insertion site for insertions and insertion-like complex events. Multiallelic records were decomposed so that each alternate allele formed an independent entry on its own row (one variant per row).

For each allele, allele count and allele number were recomputed directly from the phased genotypes rather than taken from the INFO fields: missing calls were excluded from AN, and allele frequency was AC/AN within the HPRC sample, termed as graph-internal frequency and not a population-genetic estimate.

The identical classification and anchoring pipeline was applied to *de novo* mutations from denovo-db, Seattle, WA, on the GRCh37/hg19 assembly, restricted to WGS-derived calls (records whose STUDIES SequenceType was “genome”), yielding 255,841 variants; INFO and allele-frequency fields were not used. Because the *de novo* callset is substantially smaller than the population data, variants were not size-stratified but pooled into mutation-class profiles (SNV, MNV, and simple and complex insertions and deletions), of which the multi-nucleotide class (*n* = 481) was excluded for insufficient counts (< 500).

### Generation of the control group

For each predicted Z-DNA interval identified by ZSeeker on the T2T-CHM13, GRCh38 and GRCh37 reference genomes, we generated a composition-matched control sequence on the same chromosome. Control candidates were required to match the Z-DNA interval exactly in length and to fall within a predefined tolerance range for both G + C content and CpG/GpC dinucleotide density (± 10%). Candidate control intervals were sampled at random genomic positions on the same chromosome and screened to ensure that they did not overlap any Z-DNA interval or previously assigned control. To enable efficient evaluation of sequence composition, cumulative base and dinucleotide counts were precomputed for each chromosome. For each Z-DNA interval, control candidates were iteratively sampled and tested until a suitable match was identified or a maximum number of attempts was reached. In cases where random sampling failed, a fallback strategy was applied in which candidate intervals were drawn from available non-overlapping genomic regions on the same chromosome. All accepted control intervals were non-overlapping and composition-matched to their corresponding Z-DNA interval by construction. Using this procedure, composition-matched controls were successfully assigned to 187,749 of 244,989 predicted Z-DNA intervals in GRCh37 (76.6%) and 204,721 of 274,037 intervals in T2T-CHM13 (74.7%). The remaining intervals could not be assigned a suitable control, primarily because no non-overlapping genomic region satisfying the composition-matching criteria could be identified. In analyses, where Z-DNA predictions are compared to the control sequences, only the composition-matched subset was considered.

### Intron inference

Because the reference annotation did not explicitly include intronic regions, they were inferred from exon annotations. Protein-coding genes and their associated transcripts were first identified from the GFF annotation. For each transcript with at least two annotated exons, exon coordinates were converted to 0-based half-open intervals and sorted along the transcript. Introns were then defined as the genomic intervals between consecutive exons within each transcript. All inferred intron coordinates were exported in BED format for downstream analyses.

### Allele-frequency CDS-vs-noncoding analysis

SNV allele counts and allele numbers were taken per allele as described above, and allele frequency was AF = AC/AN within the HPRC sample (a graph-internal frequency, not a population estimate). Alleles were grouped into four frequency bins - AF < 0.25% (singletons at the applied AN floor), 0.25–1%, 1–5%, and > 5% - and divided by genomic compartment: coding-sequence versus noncoding. CDS intervals were taken as the CDS features of the Liftoff v5.2 GFF annotation, converted to 0-based half-open coordinates and merged per chromosome; a SNV anchor falling within a merged CDS interval was assigned to CDS and the rest to noncoding. The primary analysis required AN ≥ 250 and retained singletons, with a minimum of 500 alleles per bin. For each compartment × AF bin, we computed the Z-DNA/control peak ratio: the mutation-anchored metaprofile over ± 500 bp was built separately for Z-DNA and matched control, each normalized to its window mean, and the ratio of mean normalized densities over ± 5 bp of the anchor was taken.

### Statistical analysis

Confidence intervals and significance were estimated using a chromosome-level block bootstrap. Correlations were assessed using Spearman’s ρ, paired comparisons using the Wilcoxon signed-rank test, group differences using the Mann-Whitney U test, and variant proximity to Z-DNA versus matched controls using discordant (McNemar) odds ratios. Multiple-testing correction was performed using the Benjamini-Hochberg procedure, and adjusted p-values are reported as q-values.

## Supplementary Material

Supplementary Files

This is a list of supplementary files associated with this preprint. Click to download.

- suppltablestatistics.xlsx
- SupplementaryMaterial.docx

## Data Availability

All analyses were performed using custom BASH scripts and Python v3.9.7 using numpy v2.0.2, pandas v2.3.1, polars v1.32.3, scipy v1.13.1, statsmodels v0.14.5, scikit-learn v1.6.1, pyranges v0.1.4, pybedtools v0.12.0, pyBigWig v0.3.24, and pyarrow v16.1.0. Figures were generated with matplotlib v3.9.4. All relevant code and scripts can be found at https://github.com/Georgakopoulos-Soares-lab/zdna_t2t_genomic_instability. The T2T-CHM13 human reference genome was downloaded from the official NCBI FTP repository (GCF_009914755.1_T2T-CHM13v2.0_genomic.fna.gz). The second release of the HPRC human haplotypes, including 464 haplotypes originating from 232 individuals, was downloaded from the official HPRC GitHub repository. The VCF file of the germline mutations generated by pangenome graph deconstruction using Wave for the second release of the human pangenome using the T2T-CHM13 as reference was obtained from the HPRC 42basepairs website (hprc-v2.0-mc-chm13.wave.vcf.gz). Reference genomes GRCh37 and GRCh38 were retrieved from https://ftp.ncbi.nlm.nih.gov/genomes/all/GCF/000/001/405/GCF_000001405.40_GRCh38.p14/GCF_000001405.40_GRCh38.p14_genomic.fna and https://ftp.ncbi.nlm.nih.gov/genomes/all/GCF/000/001/405/GCF_000001405.13_GRCh37/GCF_000001405.13_GRCh37_genomic.fna.gz respectively. Annotation files for the human T2T-CHM13 reference genome were downloaded from the official T2T-CHM13 AWS S3 repository. These included cytobands (chm13v2.0_cytobands_allchrs.bed), genomic annotations (chm13v2.0_RefSeq_Liftoff_v5.2.gff3.gz), CenSat annotations (chm13v2.0_censat_v2.1.bed), and transposable elements (chm13v2.0_RepeatMasker_4.1.2p1.2022Apr14.bed). *De novo* mutations were obtained from denovo-db, accessed in May 2026.

## References

[1] Wang AH, Quigley GJ, Kolpak FJ, Crawford JL, van Boom JH, van der Marel G, Rich A. Molecular structure of a left-handed double helical DNA fragment at atomic resolution. Nature. 1979;282:680–6. 514347 10.1038/282680a0

[2] Peck LJ, Wang JC. (1983) Energetics of B-to-Z transition in DNA. Proceedings of the National Academy of Sciences, 80, 6206–6210. 10.1073/pnas.80.20.6206 PMC394264 6578505

[3] Jovin TM, McIntosh LP, Arndt-Jovin DJ, Zarling DA, Robert-Nicoud M, van de Sande JH, Jorgenson KF, Eckstein F. Left-handed DNA: from synthetic polymers to chromosomes. J Biomol Struct Dyn. 1983;1:21–57. 6401113 10.1080/07391102.1983.10507425

[4] Krall JB, Nichols PJ, Henen MA, Vicens Q, Vögeli B. (2023) Structure and Formation of Z-DNA and Z-RNA. Molecules, 28. 10.3390/molecules28020843 PMC9867160 36677900

[5] Run Y, Tavakoli M, Zhang Y, Vasquez KM, Zhang W. Formation and biological implications of Z-DNA. Trends Genet. 2025. 10.1016/j.tig.2025.07.006. PMC12831796 40803946

[6] Sahayasheela VJ, Ooga M, Kumagai T, Sugiyama H. Z-DNA at the crossroads: untangling its role in genome dynamics. Trends Biochem Sci. 2025;50:267–79. 39875265 10.1016/j.tibs.2025.01.001

[7] Georgakopoulos-Soares I, Victorino J, Parada GE, Agarwal V, Zhao J, Wong HY, Umar MI, Elor O, Muhwezi A, An J-Y (2022) High-throughput characterization of the role of non-B DNA motifs on promoter function. Cell Genom, 2. 10.1016/j.xgen.2022.100111 PMC9105345 35573091

[8] Fang Y, Bansal K, Mostafavi S, Benoist C, Mathis D. AIRE relies on Z-DNA to flag gene targets for thymic T cell tolerization. Nature. 2024;628:400–7. 38480882 10.1038/s41586-024-07169-7 PMC11091860

[9] Beknazarov N, Konovalov D, Herbert A, Poptsova M. Z-DNA formation in promoters conserved between human and mouse are associated with increased transcription reinitiation rates. Sci Rep. 2024;14:17786. 39090226 10.1038/s41598-024-68439-y PMC11294368

[10] Shin S-I, Ham S, Park J, Seo SH, Lim CH, Jeon H, Huh J, Roh T-Y. Z-DNA-forming sites identified by ChIP-Seq are associated with actively transcribed regions in the human genome. DNA Res. 2016;23:477–86. 27374614 10.1093/dnares/dsw031 PMC5066173

[11] Liu H, Mulholland N, Fu H, Zhao K. Cooperative activity of BRG1 and Z-DNA formation in chromatin remodeling. Mol Cell Biol. 2006;26:2550–9. 16537901 10.1128/MCB.26.7.2550-2559.2006 PMC1430323

[12] Wittig B, Wölfl S, Dorbic T, Vahrson W, Rich A. Transcription of human c-myc in permeabilized nuclei is associated with formation of Z-DNA in three discrete regions of the gene. EMBO J. 1992;11:4653–63. 1330542 10.1002/j.1460-2075.1992.tb05567.x PMC557041

[13] Wong B, Chen S, Kwon J-A, Rich A. Characterization of Z-DNA as a nucleosome-boundary element in yeast Saccharomyces cerevisiae. Proc Natl Acad Sci U S A. 2007;104:2229–34. 17284586 10.1073/pnas.0611447104 PMC1892989

[14] Garner MM, Felsenfeld G. Effect of Z-DNA on nucleosome placement. J Mol Biol. 1987;196:581–90. 3681969 10.1016/0022-2836(87)90034-9

[15] Herbert A. Z-DNA and Z-RNA in human disease. Commun Biol. 2019;2:7. 30729177 10.1038/s42003-018-0237-x PMC6323056

[16] Yin C, Fedorov A, Guo H, Crawford JC, Rousseau C, Zhong X, Williams RM, Gautam A, Koehler HS, Whisnant AW, Host cell Z-RNAs activate ZBP1 during virus infections. Nature. 2025. 10.1038/s41586-025-09705-5. PMC12711578 41082924

[17] Karki R, Kanneganti T-D. ADAR1 and ZBP1 in innate immunity, cell death, and disease. Trends Immunol. 2023;44:201–16. 36710220 10.1016/j.it.2023.01.001 PMC9974732

[18] Wang G, Christensen LA, Vasquez KM. Z-DNA-forming sequences generate large-scale deletions in mammalian cells. Proc Natl Acad Sci U S A. 2006;103:2677–82. 16473937 10.1073/pnas.0511084103 PMC1413824

[19] McKinney JA, Wang G, Mukherjee A, Christensen L, Subramanian SHS, Zhao J, Vasquez KM. Distinct DNA repair pathways cause genomic instability at alternative DNA structures. Nat Commun. 2020;11:236. 31932649 10.1038/s41467-019-13878-9 PMC6957503

[20] Ravichandran S, Subramani VK, Kim KK. Z-DNA in the genome: from structure to disease. Biophys Rev. 2019;11:383–7. 31119604 10.1007/s12551-019-00534-1 PMC6557933

[21] Georgakopoulos-Soares I, Morganella S, Jain N, Hemberg M, Nik-Zainal S. Noncanonical secondary structures arising from non-B DNA motifs are determinants of mutagenesis. Genome Res. 2018;28:1264–71. 30104284 10.1101/gr.231688.117 PMC6120622

[22] Guiblet WM, Cremona MA, Harris RS, Chen D, Eckert KA, Chiaromonte F, Huang Y-F, Makova KD. Non-B DNA: a major contributor to small-and large-scale variation in nucleotide substitution frequencies across the genome. Nucleic Acids Res. 2021;49:1497–516. 33450015 10.1093/nar/gkaa1269 PMC7897504

[23] Wang G, Mouratidis I, Provatas K, Chantzi N, Patsakis M, Georgakopoulos-Soares I, Vasquez KM. (2025) ZSeeker: an optimized algorithm for Z-DNA detection in genomic sequences. Brief Bioinform, 26. 10.1093/bib/bbaf240 PMC12123511 40445004

[24] Weissensteiner MH, Cremona MA, Guiblet WM, Stoler N, Harris RS, Cechova M, Eckert KA, Chiaromonte F, Huang Y-F, Makova KD. Accurate sequencing of DNA motifs able to form alternative (non-B) structures. Genome Res. 2023;33:907–22. 37433640 10.1101/gr.277490.122 PMC10519405

[25] McGinty RJ, Sunyaev SR. Revisiting mutagenesis at non-B DNA motifs in the human genome. Nat Struct Mol Biol. 2023;30:417–24. 36914796 10.1038/s41594-023-00936-6 PMC10225297

[26] Nurk S, Koren S, Rhie A, Rautiainen M, Bzikadze AV, Mikheenko A, Vollger MR, Altemose N, Gershman A., U L, The complete sequence of a human genome. Science. 2022;376:44–53. 35357919 10.1126/science.abj6987 PMC9186530

[27] Liao W-W, Asri M, Ebler J, Doerr D, Haukness M, Hickey G, Lu S, Lucas JK, Monlong J, Abel HJ, A draft human pangenome reference. Nature. 2023;617:312–24. 37165242 10.1038/s41586-023-05896-x PMC10172123

[28] Garza Reyna A, Fuentes M, Pisetsky DS. Z-GENIE: a user-friendly R/Shiny resource for predicting Z-DNA forming regions in DNA. BMC Genomics. 2025;26:963. 41146008 10.1186/s12864-025-12148-x PMC12560280

[29] Ho PS, Ellison MJ, Quigley GJ, Rich A. A computer aided thermodynamic approach for predicting the formation of Z-DNA in naturally occurring sequences. EMBO J. 1986;5:2737–44. 3780676 10.1002/j.1460-2075.1986.tb04558.x PMC1167176

[30] Petrovič M, Bartas M, Garratt AN, Pečinka P, Dobrovolná M, Koňaříková K, Trenz O, Brázda V, Šťastný J. Z-DNA Hunter tool for straightforward detection of Z-DNA forming regions and a case study in Drosophila. NAR Genom Bioinform. 2025;7:lqaf166. 41278536 10.1093/nargab/lqaf166 PMC12634409

[31] Beknazarov N, Jin S, Poptsova M. Deep learning approach for predicting functional Z-DNA regions using omics data. Sci Rep. 2020;10:19134. 33154517 10.1038/s41598-020-76203-1 PMC7644757

[32] Umerenkov D, Herbert A, Konovalov D, Danilova A, Beknazarov N, Kokh V, Fedorov A, Poptsova M. Z-flipon variants reveal the many roles of Z-DNA and Z-RNA in health and disease. Life Sci Alliance. 2023;6:e202301962. 37164635 10.26508/lsa.202301962 PMC10172764

[33] Turner TN, Yi Q, Krumm N, Huddleston J, Hoekzema K, Stessman F, Doebley HA, Bernier A-L, Nickerson D.A. A R, and, Eichler EE. denovo-db: a compendium of human de novo variants. Nucleic Acids Res. 2017;45:D804–11. 27907889 10.1093/nar/gkw865 PMC5210614

[34] Guiblet WM, Cremona MA, Cechova M, Harris RS, Kejnovská I, Kejnovsky E, Eckert K, Chiaromonte F, Makova KD. Long-read sequencing technology indicates genome-wide effects of non-B DNA on polymerization speed and error rate. Genome Res. 2018;28:1767–78. 30401733 10.1101/gr.241257.118 PMC6280752

[35] Bacolla A, Wells RD. Non-B DNA conformations as determinants of mutagenesis and human disease. Mol Carcinog. 2009;48:273–85. 19306308 10.1002/mc.20507

[36] Bacolla A, Tainer JA, Vasquez KM, Cooper DN. Translocation and deletion breakpoints in cancer genomes are associated with potential non-B DNA-forming sequences. Nucleic Acids Res. 2016;44:5673–88. 27084947 10.1093/nar/gkw261 PMC4937311

[37] Bochalis E, Dereki I, Wang G, Sgourou A, Vasquez KM, Georgakopoulos-Soares I. (2026) Non-B DNA structures and their contributions to genetic diversity, aging, and disease. Nucleic Acids Res, 54. 10.1093/nar/gkag084 PMC12887540 41665007

[38] de Lima LG, Guarracino A, Koren S, Potapova T, McKinney S, Rhie A, Solar SJ, Seidel C, Fagen BL, Walenz BP, The formation and propagation of human Robertsonian chromosomes. Nature. 2025;647:952–61. 40993387 10.1038/s41586-025-09540-8 PMC12657243

[39] van der Maarel SM, Tawil R, Tapscott SJ. Facioscapulohumeral muscular dystrophy and DUX4: breaking the silence. Trends Mol Med. 2011;17:252–8. 21288772 10.1016/j.molmed.2011.01.001 PMC3092836

